# Supplementary material for: A giant specimen of Rhamphorhynchus muensteri and comments on the ontogeny of rhamphorhynchines
Source: PeerJ. 2025 Jan 2;13:e18587. doi: 10.7717/peerj.18587 (PMC11700493; doi:10.7717/peerj.18587)
Supplement: Supplemental Information 2 [file peerj-13-18587-s002.docx]

**Institutional Abbreviations**

**BMMS** – Bürgermeister-Müller Museum, Solnhofen, Germany

**BSPG** – Bayerische Staatssammlung für Paläontologie und Geologie, München, Germany

**CM** – Carnegie Museum of Natural History, Pittsburgh, Pennsylviana, USA

**GPIT** – Paläontologische Sammlung der Universität Tübingen, Tübingen, Germany

**GSM** – British Geological Survey Museum, Keyworth, UK

**NHMD** – Natural History Museum of Denmark, København, Denmark

**NHMUK** (formerly BMNH) – Natural History Museum, London, UK

**NMS** – National Museums Scotland, Edinburgh, UK

**SMNK** – Staatliches Museum für Naturkunde Karlsruhe, Karlsruhe, Germany

**SMNS** – Staatliches Museum für Naturkunde Stuttgart, Stuttgart, Germany

**YPM** – Yale Peabody Museum, New Haven, Connecticut, USA (VPPU designates vertebrate paleontology specimens formerly held at Princeton University, Princeton, New Jersey, now held at YPM.)

**ZDM** – Zigong Dinosaur Museum, Zigong, Sichuan, China

BSPG 1889 XI 1

BMMS 3A

CM 11431, CM 11434

GPIT RE/7321

GSM 3166

NHMD 1891.738

NHMUK R 231, NHMUK R 2786, NHMUK PV OR 37002, NHMUK PV OR 37003, NHMUK 4121, NHMUK PV OR 42737, NHMUK 43004

NMS G.2021.6.1-4

SMNK PAL 6596

SMNS 55886

YPM VP 1778

ZDM T8001
