# Supplementary material for: A giant specimen of Rhamphorhynchus muensteri and comments on the ontogeny of rhamphorhynchines
Source: PeerJ. 2025 Jan 2;13:e18587. doi: 10.7717/peerj.18587 (PMC11700493; doi:10.7717/peerj.18587)
Supplement: Supplemental Information 1 — A photogrammetric model of NHMUK PV OR 37002 in 3D taken before the recent repreparation work to document the state of the specimen before it was worked on. [file peerj-13-18587-s001.pdf]

Generated with Agisoft PhotoScan
